# Surgical outcomes of coronal shear fractures of the capitellum and trochlea: a retrospective study of Dubberley classification-guided treatment

**DOI:** 10.1515/med-2025-1366

**Published:** 2026-02-25

**Authors:** Qing Yang, Hailun Gu, Dapeng Zhou, Gengqi Wang, Qiugen Wang

**Affiliations:** Department of Orthopedics, Yueyang Hospital of Integrated Traditional Chinese and Western Medicine, Shanghai University of Traditional Chinese Medicine, Shanghai, China, 110 Ganhe Road, Hongkou District, Shanghai, 200437, P.R. China; Department of Orthopedics, Shengjing Hospital, China Medical University, Shenyang, Liaoning Province, P.R. China; Department of Orthopedics, General Hospital of Northern Theater Command of the PLA, Shenyang, Liaoning Province, P.R. China

**Keywords:** fracture, distal humerus, classification, surgery, functional outcome

## Abstract

**Objectives:**

To investigate the surgical outcomes of coronal shear fractures of the capitellum and trochlea (CSFCT) guided by the Dubberley classification.

**Methods:**

A retrospective analysis was conducted on patients with CSFCT between January 2015 and December 2023. Demographic information, surgical details, functional recovery outcomes, and postoperative complications were recorded and compared across fracture subgroup.

**Results:**

39 patients were included (mean age: 44.3 ± 15.5 years) with a mean follow-up of 38.2 ± 12.0 months. All patients underwent surgery via an extended lateral approach using various hardware. Postoperatively, the mean extension deficit was - 0.9°, mean flexion was 126.4°, and mean flexion-extension arc was 125.4°. The mean Mayo Elbow Performance Scores (MEPS) was 87.9 (66.7 % excellent, 30.8 % good, 2.5 % fair). Dubberley type 2 and A fractures had higher MEPS than type 3 and B, respectively (both p<0.01). Postoperative complications included two elbow stiffness, two implant prominence, and one avascular necrosis case.

**Conclusions:**

Under the guidance of the Dubberley classification, surgical treatment for patients with CSFCT has achieved greater efficacy, favorable outcomes, and low complication rates.

## Introduction

Coronal shear fractures of the capitellum and trochlea (CSFCT) represent a rare subset of elbow injuries, accounting for only 0.5–1 % of all elbow fractures [1], 2]. Typically caused by axial loading during falls, these fractures produce coronal shear fragments in the capitellum and trochlea. In more severe cases, the fracture may extend to involve the posterior condyle and lateral column [3], which aligns with the AO/OTA classification of type B3.3 [4]. This classification corresponds to the Dubberley type 2 (simple) or type 3 (comminuted) patterns, first established in 2006 [5]. Given the complex anatomy of the elbow joint, achieving anatomical reduction and ensuring rigid fixation are pivotal to preventing postoperative complications and promoting optimal functional recovery.

Regarding surgical interventions, while lateral or extended lateral approaches are commonly used, they are associated with a relatively high complication rate [6]. Anterolateral exposure provides direct access to the anterior aspect of the joint, whereas posterior approaches are particularly advantageous for managing column comminution [7]. Compared with transolecranon techniques [8], extended lateral approaches tend to yield superior functional outcomes [9]. Previous studies have documented the use of headless compression screws (HCSs) for simple fractures [10], [11], [12], antiglide plates for comminuted fragments [13], 14], and locking plates for fractures involving the lateral column [13], 15]. Despite significant advancements, surgical treatment of CSFCT remains challenging: reported complications include an overall rate of 19.8 %, symptomatic implants in 10.4 % of cases, and elbow stiffness in 6 % of patients [16], [17], [18]. Moreover, no consensus has been reached on the most appropriate surgical strategies for different fracture subtypes. Against this backdrop, this study aims to systematically evaluate the efficacy of surgical strategies for CSFCT treatment, guided by the Dubberley classification.

## Patients and methods

### Study population

This retrospective study enrolled patients with CSFCT who underwent surgery at our trauma center between January 2015 and December 2023. Medical records of all fractures were systematically reviewed to identify cases involving both the capitellum and trochlea. Exclusion criteria were: (1) skeletal immaturity (<18 years), (2) open fractures, (3) pathological fractures, and (4) multiple upper limb injuries. Of the 42 initially eligible patients, three were excluded due to incomplete follow-up data. The final cohort comprised 39 patients with complete clinical records.

For each case, demographic data, injury mechanisms, associated injuries, surgical approaches, fixation methods, and hardware selection were documented. Two experienced surgeons classified CSFCT using the Dubberley system [5] based on pre- and postoperative imaging (including X-rays and computed tomography [CT] scans), dividing the fractures into type 2 and type 3, each with subtypes A and B. Regarding the reliability of the Dubberley classification, a post hoc inter-rater analysis (involving the two surgeons, using Cohen’s kappa) showed substantial agreement (κ=0.86, 95 % CI: 0.72–0.98), confirming its reliability.

**Ethical statement:** This study was approved by the Institutional Ethics Committee of Yueyang Hospital of Integrated Traditional Chinese and Western Medicine, Shanghai University of Traditional Chinese Medicine (No. 2024–229), and conducted in accordance with the principles of the 1964 Declaration of Helsinki. All participants provided written consent for the publication of their X-rays and photographs.

### Surgical procedures

All surgical procedures were performed under general anesthesia with patients in the supine position. An extended lateral elbow approach, centered on the lateral epicondyle, was used to access the surgical site; a medial approach was added if necessary. Dissection was performed carefully along the plane between the anconeus (posterior) and extensor carpi ulnaris (anterior) muscles. Elevating the extensor tendon complex anteromedially exposed the anterior joint capsule, which allowed direct visualization of anterior articular fragments and posterior condylar elements. Under direct vision, all fractures underwent open reduction and internal fixation (ORIF).

**Figure 1:**
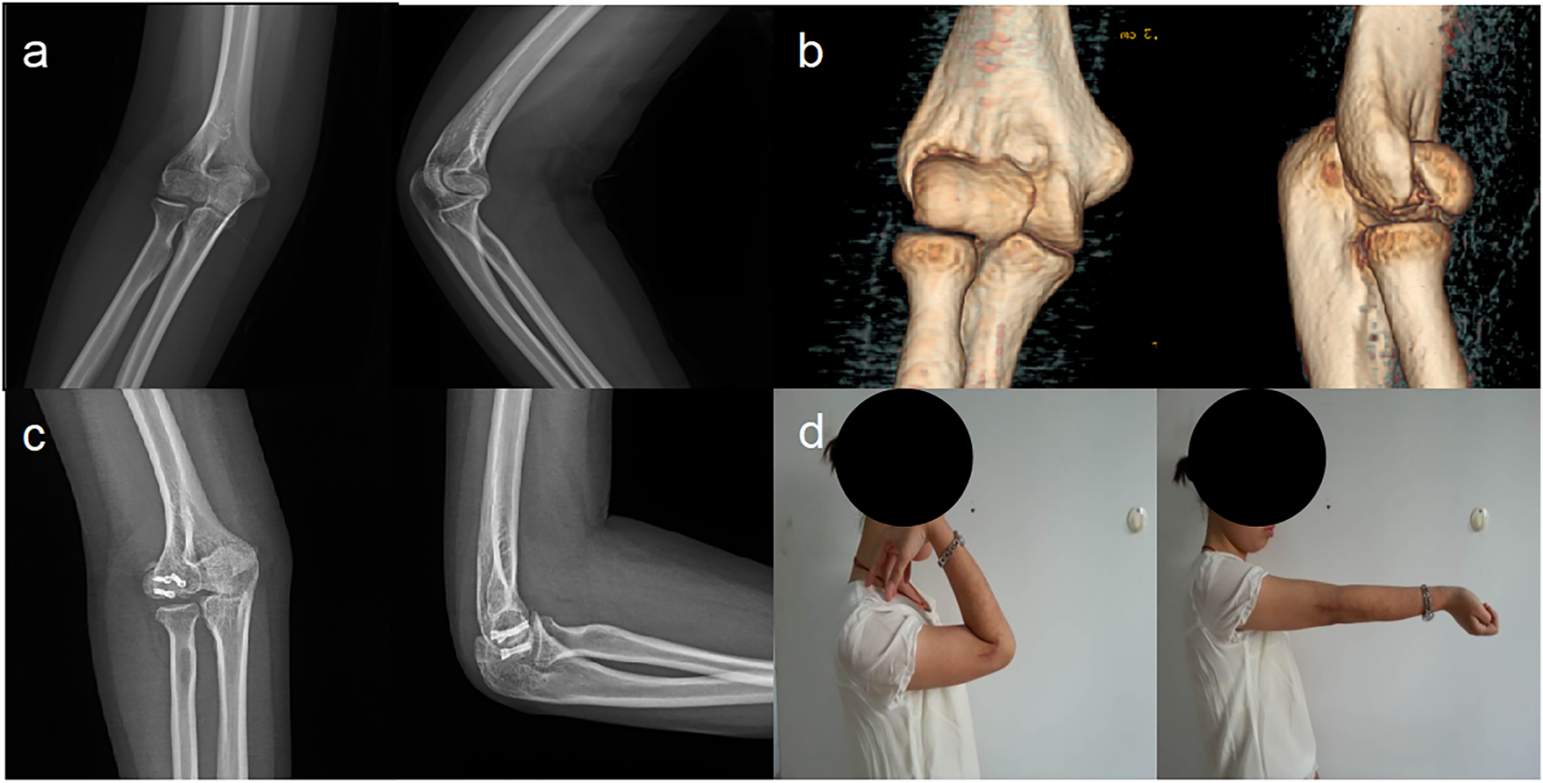
Dubberley type 2 fracture: a A 30-year-old female had a coronal shear fracture of distal humerus (Dubberley 2A) with preoperative X-ray images of distal humerus. b Preoperative CT images showed intact fragment of capitellum and trochlea. c Postoperative X-ray images showed three headless compression screws for fracture fixation. d Functional outcome was evaluated to be good at last visit at least 1 year following-up.

**Figure 2:**
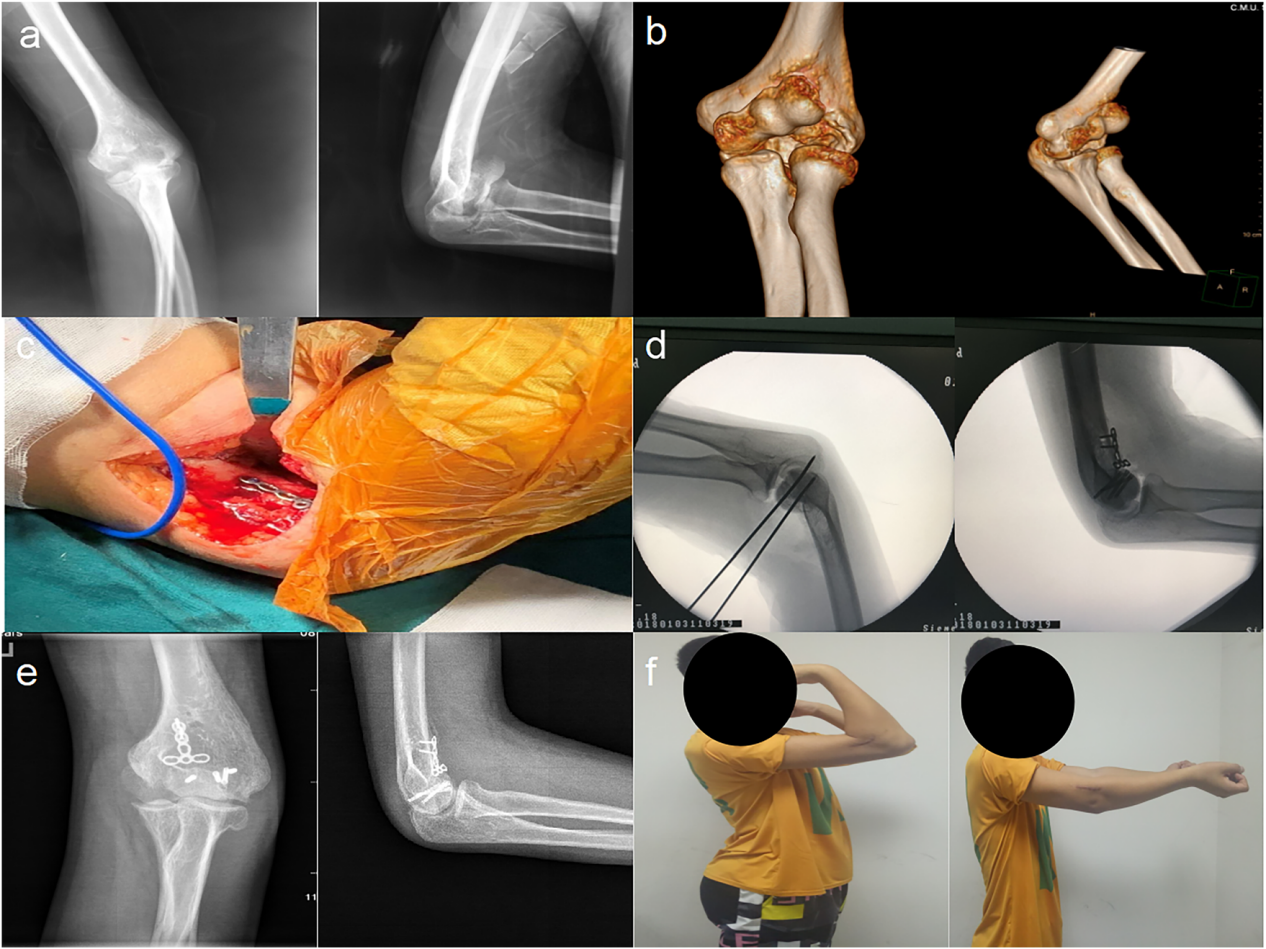
Dubberley type 3A fracture: a A 24-year-old male had a coronal shear fracture of distal humerus (Dubberley 3A) with preoperative X-ray images of distal humerus. b Preoperative CT images showed seperated fragments of capitellum and trochlea. c Extended lateral provided an optimal window to exposure and a mini T-shaped plate was used to anti-resist the coronal shear forces of distal humerus. d Intraoperative images of C-arm guided fracture reduction and internal fixatoin. e Postoperative X-ray images showed good internal fixation with three headless compression screws and a mini T-shape plate for fracture fixation. f At final following-up the young man had good range of motion and returned to former work.

**Figure 3:**
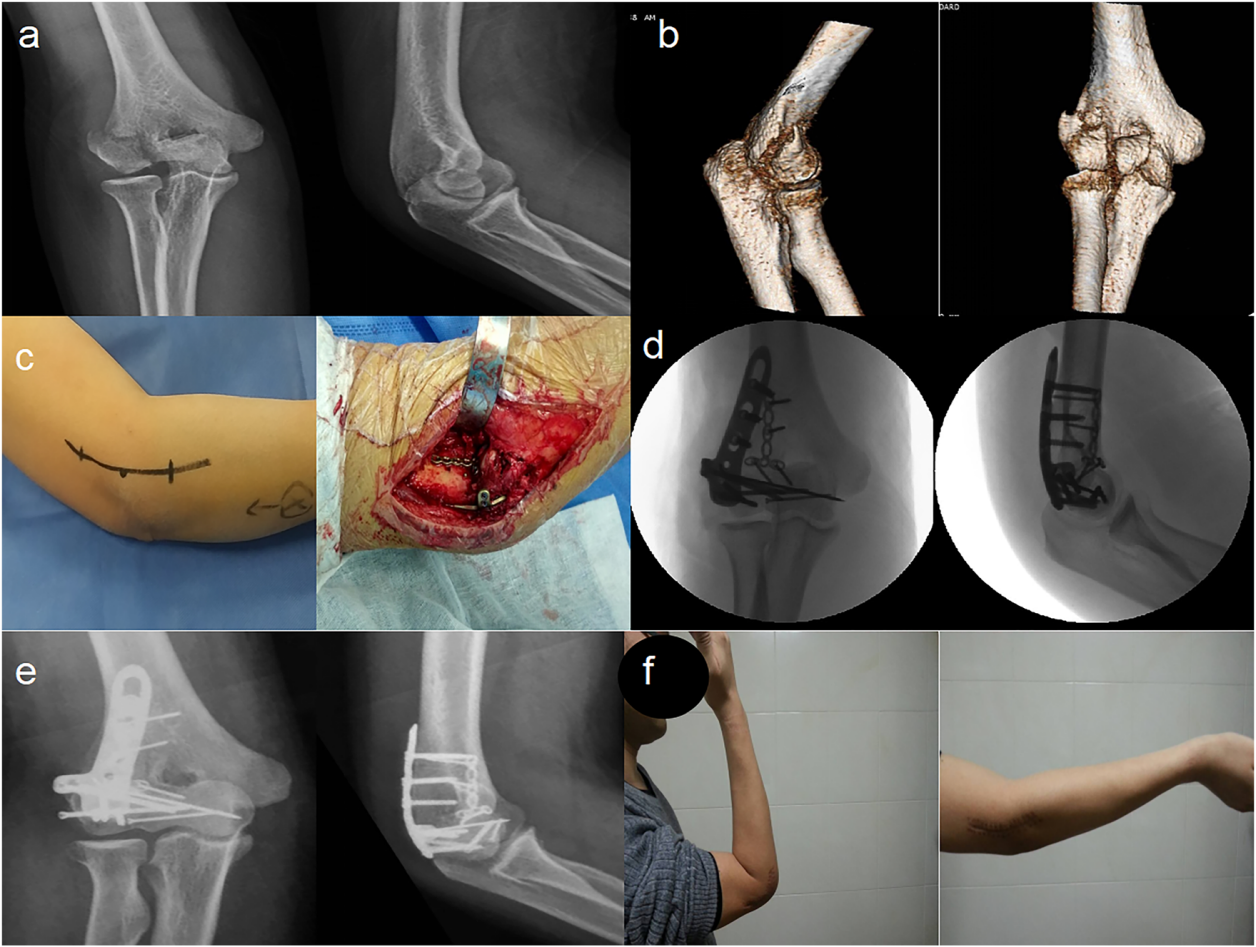
Dubberley type 3B fracture: a A 37-year-old male had a coronal shear fracture of distal humerus (Dubberley 3B) with preoperative X-ray images of distal humerus. b Preoperative CT images showed seperated fragments of capitellum and trochlea and comminution of posterior condylar. c Extended lateral incision was applied. f Intraoperative image of a micro-T-shape plate and a locking compression plate was showed. d Intraoperative C-arm images were evaluated. e postoperative X-ray images showed anatomic reduction and rigid fixation using all surgical techniques. f The patient was satisfied with the final outcome at his 1-year visit, and there was no significant influnce in his noraml activity.

Per the study’s surgical strategies (tailored to Dubberley fracture subtypes), simple CSFCT fractures (Dubberley type 2) were anatomically reduced and stabilized with 3.0–4.0 mm headless compression screws (HCSs; partially threaded, cannulated design) for articular reconstruction (Figure 1). Comminuted fractures (Dubberley type 3) were treated with 2.7–3.5 mm HCSs (for primary articular fragment fixation) and 1.0–1.2 mm Kirschner wires (KWs; smooth, straight type) for auxiliary fragment stabilization. If joint assessment revealed coronal instability, a 2.0 mm mini T-shaped plate (m-TP; titanium alloy, pre-contoured) was added to enhance fixation strength in the coronal plane (Figure 2). For Dubberley subtype B fractures (characterized by column comminution), a 3.5 mm posterolateral locking compression plate (pl-LCP; titanium alloy, variable-angle locking holes) was used to restore column integrity (Figure 3). Concomitant epicondylar fractures and ligamentous injuries were also addressed (e.g., lateral collateral ligament repair with 2.0 mm suture anchors) to optimize functional outcomes.

### Postoperative care

Postoperatively, all patients wore an adjustable elbow brace (90° flexion) for 4–6 weeks, with passive ROM exercises at 2 weeks (physical therapy-guided) and active exercises after brace removal. Oral indomethacin (25 mg tid) was given for 6 weeks to prevent HO, with adverse reaction monitoring. Follow-up: 2 weeks (suture removal, brace adjustment), 6 weeks (healing check, brace decision), 3/6/12 months; radiographs at each visit to assess reduction, consolidation, complications. HO was diagnosed via radiography (obvious) or CT (subtle) per Brooker classification [19]. Strengthening exercises started after radiographic union (bony trabeculation, 3–6 months). Elbow stiffness: >30 % ROM reduction vs. contralateral elbow; AVN (capitellum/trochlea) diagnosed via MRI [20]. Mobility measured by ROM, functional outcomes via MEPS [21]. Postoperative complications (infections, neurovascular injuries, AVN) were monitored. Revision surgery indicated for symptomatic HO (refractory ROM limitation), severe stiffness (ROM <90° post-3-month rehabilitation), AVN-related collapse, or hardware failure.

### Statistical analysis

Statistical analyses were performed using SAS software (version 9.4; SAS Institute Inc., Cary, NC, USA). Continuous variables are presented as mean ± standard deviation (x̄ ± s). Elbow ROM and MEPS values were compared between fracture types using independent-sample t-tests. Categorical variables were analyzed with Pearson’s chi-squared test. Revision-free rates were estimated using the Kaplan-Meier method at 12 and 24 months. Normality and other statistical test assumptions were verified (via Shapiro-Wilk test for normality, Levene’s test for homogeneity of variance) before analysis, and all tests were two – sided. A strict significance level of p<0.01 was adopted for all statistical comparisons.

## Results

This study enrolled 39 patients (20 males, 19 females; mean age 44.3 ± 15.5 years, range 18–75 years). Fractures involved the right arm (20 cases) and left arm (19 cases); primary injury mechanism were falls (n=33), plus one traffic accident and one heavy glass trauma, all fractures closed. Concomitant injuries (15 cases): five medial/lateral epicondylar fractures, two radial head fractures, one collateral ligament lesion, two olecranon fractures, two elbow dislocations with lateral epicondylar fractures, two polytrauma. Surgery was performed 3.7 ± 1.2 days post-injury (range 3–10 days), with mean operative time 70.4 ± 17.7 min (range 50–120 min). CSFCT were classified via Dubberley system as type 2A (30.8 %, n=12), type 2B (7.7 %, n=3), type 3A (20.5 %, n=8), type 3B (41.0 %, n=16); mean follow-up was 38.2 ± 12.0 months (range 24–72 months).

Surgical strategies by Dubberley type: type 2A (n=12) used HCSs with KWs (fragment compression, coronal stability); type 2B (n=3, posterior condylar comminution) combined pl-LCPs (column reconstruction) with HCSs with KWs; type 3A (n=8) applied anterior m-TPs (shear force neutralization, coronal stability) in 6 cases; type 3B (n=16, challenging fixation) primarily used pl-LCPs, with additional m-TPs (coronal stability) in 6 cases. Suture anchors repaired associated epicondylar fractures/ligaments in 6 cases.

The mean extension range was −0.9° (range: −20° to 15°), mean flexion was 126.4° (range: 120°–135°), and the average flexion-extension arc was 125.4° (range: 100°–140°). When comparing fracture types, type 2 fractures outperformed type 3 in extension (4.1 ± 4.2° vs. −4.1 ± 5.7°, p<0.01), flexion (129.3 ± 5.6° vs. 124.5 ± 4.3°, p<0.01), and flexion-extension arc (133.5 ± 4.0° vs. 120.4 ± 8.2°, p<0.01). At the subtype level, type A fractures demonstrated better extension than type B fractures (1.85 ± 6.5° vs. −3.89 ± 5.2°, p<0.01), though no significant differences were observed in flexion or flexion-extension arc between the two subtypes (p>0.01; Table 1).

**Table 1:** Comparison of functional outcomes in different Dubberley types.

Functional outcomes	Total	Type 2	Type 3	3 vs. 2a	Type A	Type B	B vs. A
Extension	−0.9 ± 6.5	4.1 ± 4.2	−4.1 ± 5.7	<0.0001	1.85 ± 6.5	−3.89 ± 5.2	0.0045
Flexion	126.4 ± 5.3	129.3 ± 5.6	124.5 ± 4.3	0.0045	127.0 ± 5.5	125.7 ± 5.3	0.4499
Arc	125.4 ± 9.4	133.5 ± 4.0	120.4 ± 8.2	<0.0001	128.9 ± 8.6	121.8 ± 9.1	0.0171
MEPS	89.1 ± 7.2	94.7 ± 4.0	85.6 ± 6.5	<0.0001	93.3 ± 4.1	84.7 ± 7.2	<0.001

Functional outcomes expressed by mean ± SD MEPS, Mayo Elbow Performance Scale.

**Figure 4:**
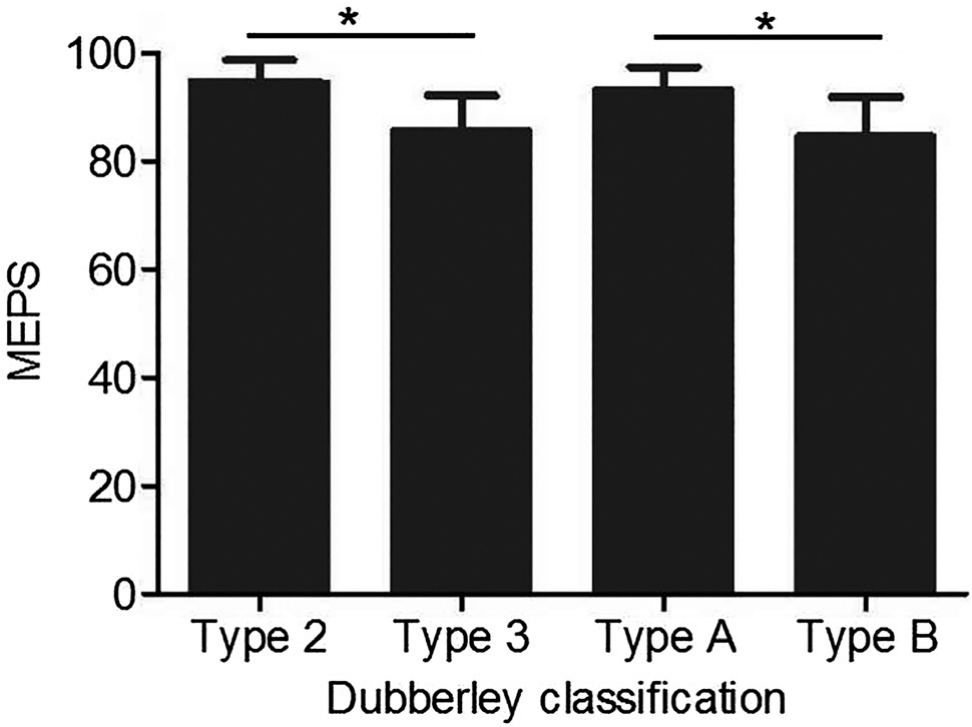
Functional scores correlated with Dubberley classification: MEPS was showed in different Dubberley types, and it was significant when type 2 vs. Type 3, and type A vs. Type B (p<0.01). MEPS, Mayo Elbow Performance Scale.

The mean MEPS was 87.9 points (range: 70–100), with outcomes distributed as follows: 66.7 % (26 patients) achieved excellent results, 30.8 % (12 patients) good, and 2.5 % (1 patient) fair. Type 2 fractures had a significantly higher mean MEPS than type 3 fractures (94.2 ± 6.1 vs. 85.6 ± 6.5, p<0.01), and type A fractures had a higher mean MEPS than type B fractures (93.3 ± 4.1 vs. 84.7 ± 7.2, p<0.01); among type 3 2fractures, subtype 3B had an average MEPS of 83.1 ± 6.6. Excellent outcomes were also more frequent in type 2 vs. type 3 fractures (14 vs. 12 cases, p<0.01) and in type A vs. type B fractures (20 vs. other cases, p<0.01; Table 2, Figure 4).

**Table 2:** Comparison of MEPS in different Dubberley types.

MEPS	Total	Type 2	Type 3	3 vs. 2	Type A	Type B	B vs. A
Excellent^a^	26	14	12	<0.01	20	6	<0.01
Good	12	1	11	–	0	12	–
Fair	1	0	1	–	0	1	–

^a^Pearson’s Chi-squared test. MEPS, Mayo Elbow Performance Scale. Excellent (90–100 points), good (75–89 points), fair (60–75 points).

All fractures achieved union, with no cases of nonunion or delayed union. Throughout follow-up, there were no instances of infection, nerve injury, degenerative arthritis, or HO. Postoperative adverse events were limited to: 2 cases requiring hardware revision (at 15 and 18 months postoperatively, due to implant prominence), two patients undergoing closed release (at 6 and 9 months postoperatively, for elbow stiffness, with subsequent ROM restoration), and 1 case of AVN accompanied by screw loosening (at 18 months postoperatively, with pain resolved following implant removal). Cumulatively, three revision surgeries were performed by 12 months, and five by 24 months. The Kaplan-Meier-estimated revision-free rates were 92.3 % (95 % CI: 85.1–99.5 %) at 12 months and 87.2 % (95 % CI: 78.5–99.5 %) at 24 months.

## Discussion

This retrospective study demonstrated improved functional outcomes and low complication rates in the treatment of CSFCT using Dubberley classification-guided surgical strategies: an extended lateral approach, with a medial approach added if needed, provided optimal exposure for fixation, intraoperative stability assessment was critical, and m-TPs enhanced coronal plane stability. Additionally, the coordinated use of precise Dubberley classification, rigid fixation, and early functional exercises addressed current surgical limitations for CSFCT, which ultimately yielded favorable elbow range of motion and high MEPS results.

An optimal surgical approach facilitates fracture exposure, enables rigid fixation, and reduces complications [16], 22]. While AO/OTA-based surgery is standard [10], approach choice significantly impacts outcomes. The extended lateral approach is most common [6], providing anterior exposure similar to the anterolateral approach and being particularly beneficial for trochlear involvement [7]. Posterior transolecranon approaches typically yield lower MEPS scores [8]. Guidelines recommend matching approach to fracture complexity: extended lateral for posterior comminution and transolecranon for Dubberley type 3 fractures [23], 24]. Our 39-case study shows the extended lateral approach enables effective exposure and stabilization, achieving good MEPS scores with manageable complications. For severe trochlear comminution, adding a medial approach improves visualization, accuracy, and treatment outcomes [10], 25], 26], which balances surgical feasibility and functional results.

Surgical strategies for CSFCT follow anatomical reduction, rigid fixation, and early mobilization [9], 10], 17], 22], but Dubberley type 3 fractures remain challenging due to small articular fragments, high shear forces, and limited fixation [12], 13], 16], 17], which makes the Dubberley classification system (guiding personalized planning [17]) critical; the antiglide plate technique (first introduced by Milan [27]) addresses these challenges by neutralizing shear forces for isolated capitellum/trochlea fractures, with a clinical study [14] reporting good outcomes in 42 patients and a biomechanical study [13] confirming its fixation strength (with HCSs) matches pl-LCP in Dubberley 2B fractures, and in our study, we optimized this technique for Dubberley type 3 fractures: 50 % used m-TP as an antiglide plate (with KWs and HCSs) for coronal stability, which when paired with LCP-based column restoration achieved 100 % rigid fixation and early mobilization, ultimately leading to exceptional results, including all patients having favorable outcomes, minimal hardware complications, and functional data (mean flexion 126.4°, near-complete extension - 0.9°, MEPS 87.9), and compared to broader trends, m-TP-enhanced fixation reduced revisions and adverse events, linking technical success to patient outcomes.

Our study achieved excellent functional outcomes: mean flexion was 126.4°, near-complete extension (-0.9°), and range of motion arc was 125.4°, which surpasses most prior studies [4], 7], 16], 25] except one [26], with a mean MEPS of 87.9 that exceeds 84.9 from one study [16] but is lower than 96.3 from another [27]; notably, our cohort excluded Dubberley type 1 fractures and included more type 3 fractures, which makes these results more meaningful for complex cases. We also had a lower complication rate than previous studies [4], 6], 17], 25], 27], and this is attributed to rigid fixation with focused coronal stability that enabled early mobilization: all fractures united without nonunion, infection, nerve injury, dislocation, or HO; revision rate dropped from 13.8 % [6] and 19.8 % [18] to 5.1 %, with two hardware prominence cases resolved via implant removal; elbow stiffness affected 5.1 % (two patients), which is well below the 6–28.6 % prior range [17], 18], and both patients regained full motion after release; AVN occurred in 2.6 % (one patient) vs. 4.5 % in literature [6], this case was asymptomatic until screw loosening [28], with radiographic AVN signs preceding symptoms, and all complications were resolved after hardware removal at final follow-up.

Current fracture classification systems cannot cover all types due to fractures’ polymorphism and complexity; the Dubberley classification is more suitable for these fractures than the Bryan and Morrey system [10], though its prognostic efficacy lacks comprehensive validation. One study [29] found significant MEPS differences between Dubberley subtypes A and B, while a review [9] and a modified Dubberley study [30] reported no significant differences in outcomes/ROM by subtype or type (1–4). In contrast, a Dubberley 3B study [31] noted worsening outcomes from type 1–3/A – B, with worse MEPS in group B (≥3 fragments). Our results show type 2 fractures had better extension/flexion/arc and higher MEPS than type 3, with subtype A/B differences only in extension and MEPS. These align with prior trends [29], 31], 32], possibly due to case/grouping differences, so high-quality comparative studies are needed for validation.

This study has several limitations. First, the small sample size (due to this fracture’s low incidence) limits statistical power for comprehensive comparisons, prevents controlling for influential concomitant injuries, requires probabilistic categorization of unclear fractures (risking distorted outcomes), and minor intra-group treatment variations may affect functional results and complicate analyses. Second, the retrospective design carries risks of selection or information bias, which can hinder accurate interpretation of the study results. Third, the absence of a control group means we cannot draw definitive conclusions about whether the proposed treatment is superior to alternative approaches.

## Conclusions

Guided by the Dubberley classification, which is more effective for CSFCT categorization than alternatives like Bryan and Morrey, surgical strategies improved CSFCT functional outcomes. Despite CSFCT challenges including polymorphic morphology, classification limits, shear forces and especially Dubberley type 3 fractures, optimal results are achievable via integrated precise classification, rigid fixation such as m-TP with LCP, and early exercise. These findings support the Dubberley-guided approach as a valuable CSFCT framework, while noting larger, high-quality studies are needed for validation and to address limitations.
